# Is serum biotinidase enzyme activity a potential marker of perturbed glucose and lipid metabolism?

**DOI:** 10.1002/jmd2.12168

**Published:** 2020-10-06

**Authors:** Patrick Forny, Patricie Burda, Peter Bode, Marianne Rohrbach

**Affiliations:** ^1^ Division of Metabolism and Children's Research Center University Children's Hospital Zurich Zurich Switzerland; ^2^ Institute of Surgical Pathology University Hospital Zurich Zurich Switzerland

**Keywords:** biotinidase, fatty acid synthesis, gluconeogenesis, glycogen storage disease, GSD Ia, GSD IV

## Abstract

Glycogen storage diseases (GSDs) belong to the group of inborn errors of carbohydrate metabolism. Hepatic GSDs predominantly involve the liver and most present with hepatomegaly. Biochemically they show known disturbances in glucose and fatty acids metabolism, namely fasting hypoglycaemia and increased triglycerides. Additionally, increased biotinidase (BTD) enzyme activity has been shown to be associated with many GSD types, whereas the mechanism by which BTD enzyme activity is altered remains unknown so far. We aimed to delineate changes in gluconeogenesis and fatty acid synthesis, potentially explaining raised BTD enzyme activity, by using liver (specimens from 2 patients) and serum samples of GSD Ia and GSD IV patients. By expression analysis of genes involved in gluconeogenesis, we ascertained increased levels of phosphoenolpyruvate carboxykinase and fructose‐1,6‐biphosphatase, indicating an increased flux through the gluconeogenic pathway. Additionally, we found increased gene expression of the biotin‐dependent pyruvate and acetyl‐CoA carboxylases, providing substrate for gluconeogenesis and increased fatty acid synthesis. We also observed a significant linear correlation between BTD enzyme activity and triglyceride levels in a cohort of GSD Ia patients. The results of this pilot study suggest that enhancement of BTD activity might serve the purpose of providing extra cofactor to the carboxylase enzymes as an adjustment to disturbed glucose and fatty acid metabolism. Future studies involving a higher number of samples should aim at confirming the here proposed mechanism, which extends the application of BTD enzyme activity measurement beyond its diagnostic purpose in suspected GSD, and opens up possibilities for its use as a sensor for increased gluconeogenesis and fatty acid synthesis.

**Synopsis**

Biotinidase enzyme activity can be used as a diagnostic screening tool for glycogen storage disease and might be suitable for detection of increased gluconeogenesis and fatty acid synthesis.

## 1 INTRODUCTION

Glucose is the most important source of energy, which must be constantly available, and its level is meticulously controlled to maintain normoglycaemia. Following carbohydrate intake, glucose is directly broken down via glycolysis, while it must be newly generated via gluconeogenesis or glycogenolysis during times of fasting. Glycolysis is a multi‐step process to produce pyruvate, which is oxidized to acetyl‐CoA and further metabolized to malonyl‐CoA by acetyl‐CoA carboxylase (ACACA; EC 6.4.1.2) in the process of fatty acid synthesis. In the reverse process, called gluconeogenesis, pyruvate is metabolized to oxaloacetate by pyruvate carboxylase (PC; EC 6.4.1.1)^1^ and further processed by the two key enzymes of gluconeogenesis: phosphoenolpyruvate carboxykinase (PCK1 (cytosolic) and PCK2 (mitochondrial); EC 4.1.1.32) and fructose‐1,6‐biphosphatase (FBP1; EC 3.1.3.11).^2^, ^3^

Storage of glucose as energy source is enabled by the build‐up of glycogen, a polysaccharide consisting of linked glucose molecules via linear α(1 → 4) and branched α(1 → 6) glycosidic bonds. Deficiencies of glycogen synthesis and breakdown cause glycogen storage disorders (GSDs), a heterogenous group of inherited disorders of carbohydrate metabolism.^4^ In this work, we studied the following two hepatic forms of GSD: GSD Ia, caused by deficient glucose‐6‐phosphatase (G6PC; EC 3.1.3.9) and GSD IV, caused by deficiency of the 1,4‐alpha‐glucan‐branching enzyme (GBE1; EC 2.4.1.18). The former is characterized by hepatomegaly, lactic acidosis, and hyperlipidaemia,^5^ whereas the latter typically shows progressive liver disease, including hepatomegaly (accumulating polyglucosan), failure to thrive, and muscular hypotonia.^6^ Hypoglycaemia is a cardinal symptom of GSD Ia, due to disrupted gluconeogenesis and impaired glycogen breakdown, whilst in GSD IV hypoglycaemia only occurs during end stage cirrhosis.^7^

In GSD Ia, the accumulating glucose‐6‐phosphate is shunted to glycolysis, leading to an increase of acetyl‐CoA and thereby raised lipogenesis and malonyl‐CoA through the action of ACACA, whereby the latter metabolite inhibits carnitine palmitoyltransferase I, leading to a further increase of triglycerides in the plasma of patients.^8^ In GSD IV on the other hand, we propose that due to the insoluble glycogen polymer, gluconeogenesis is chronically upregulated as a compensatory mechanism, whereby PC facilitates the first step by producing oxaloacetate.

We analyzed liver tissues of a GSD Ia and a GSD IV patient for the expression of *BTD*, *PCK1*, *PCK2*, *FBP1*, *ACACA*, *PC*, and assessed BTD enzyme activity in serum of these patients. We complemented these investigations with BTD enzyme activity measurements in two non‐GSD patient cohorts with a normal glycogen storage pathway, aiming to study the contribution of increased gluconeogenesis and an altered fatty acid oxidation pathway to elevated BTD enzyme activity. First, we investigated a cohort of diabetes mellitus type 1 patients, who are known to have upregulated gluconeogenesis.^9^ Second, patients on a ketogenic diet were studied, where fatty acid oxidation provides ATP as energy source to sustain gluconeogenesis and acetyl‐CoA to activate pyruvate carboxylase.^10^

**FIGURE 1:**
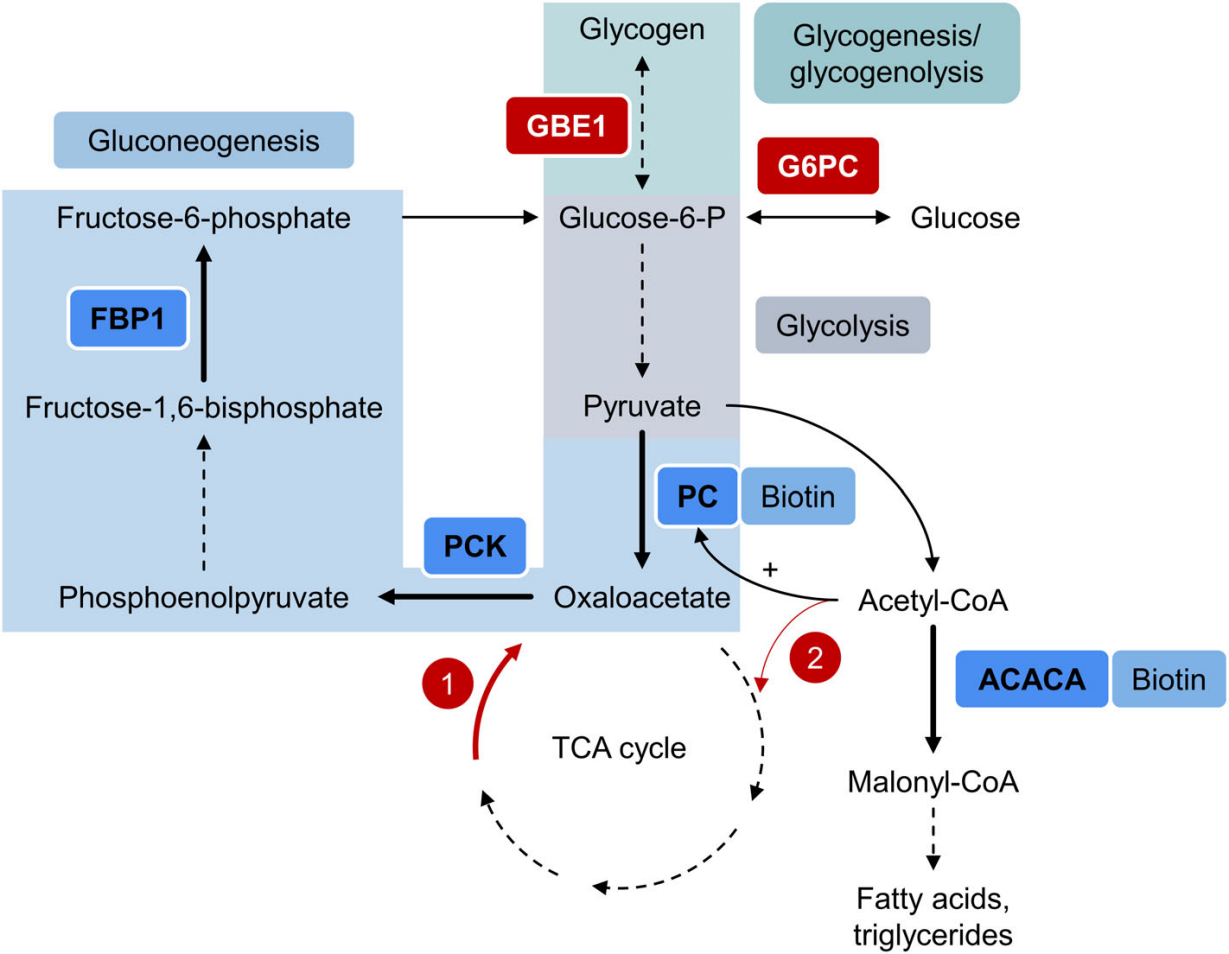
Proposed dysregulation of glucose and fatty acid metabolism. Solid black arrows are direct biochemical reactions. Dashed black arrows are reactions with intermediary steps. The four black bold arrows represent reactions for which the gene of the respective enzyme (acetyl‐CoA carboxylase, ACACA; pyruvate carboxylase, PC [the latter two depicted with biotin cofactor]; fructose‐1,6‐biphosphatase, FBP1; phosphoenolpyruvate carboxykinase, PCK) was shown to be upregulated in this study. Red arrows depict pathways particularly relevant to our mechanistic proposal and indicate (1) provision of oxaloacetate to feed into gluconeogenesis (red bold arrow), and (2) accumulating acetyl‐CoA levels because of tricarboxylic acid (TCA) cycle cataplerosis and consequently lack of oxaloacetate to form citrate with acetyl‐CoA (red fine arrow). ACACA, acetyl‐CoA carboxylase; GBE1, 1,4‐alpha‐glucan‐branching enzyme; G6Pase, glucose‐6‐phosphatase; PC, pyruvate carboxylase; PCK, phosphoenolpyruvate carboxykinase; FBP1, fructose‐1,6‐biphosphatase; “+” indicates activation of PC by acetyl‐CoA

We hypothesize, that in both GSD types one of the biotin‐requiring carboxylases PC or ACACA is upregulated, hence the requirement for their cofactor biotin is increased (Figure 1). We further hypothesize, that biotinidase (BTD; EC 3.5.1.12) enzyme activity is raised as a response to the increased requirement of biotin. While we have previously observed increased biotinidase enzyme activity in hepatic GSD patients,^11^ the presented work investigates gene expression of enzymes involved in gluconeogenesis and fatty acid metabolism to investigate if the proposed dysregulations are present in GSD Ia and GSD IV.

## 2 PATIENTS AND METHODS

### 2.1 Liver and serum samples

Liver samples of a GSD Ia and a GSD IV patient (both paediatric) were harvested for diagnostic purposes and then used for the expression analyses outlined in this study. Due to limited availability of other liver samples, the study was restricted to these GSD subtypes. Two anonymised control liver samples from healthy donors without further clinical information were retrieved from the Centralized Tissue‐Biobank of the University Hospital Zurich.

Serum samples of the two GSD patients mentioned above and a group of diabetes mellitus type 1 patients (n = 5; median age: 15.5 years, range 14.2‐16.2 years), epilepsy patients on ketogenic diet (n = 4; median age: 3.0 years, range: 0.4‐5.7 years) as well as of a cohort of enzymatically and/or molecularly confirmed GSD Ia patients (n = 7; median age: 9.7 years, range 0.2‐46.8 years) were obtained for the study of BTD enzyme activity by routine blood collection during clinic visits. The patients on ketogenic diet were treated with different regimes of antiepileptic drugs at the time of biotinidase enzyme activity determination (patient 1: clonazepam; patient 2: clobazam, phenytoin, lamotrigine; patient 3: valproate, topiramate, clobazam; patient 4: phenobarbital, topiramate, lamotrigine). The antiepileptic medication remained unchanged for all measurements of biotinidase enzyme activity.

### 2.2 Patients

#### 2.2.1 Case 1 GSD IV

**FIGURE 2:**
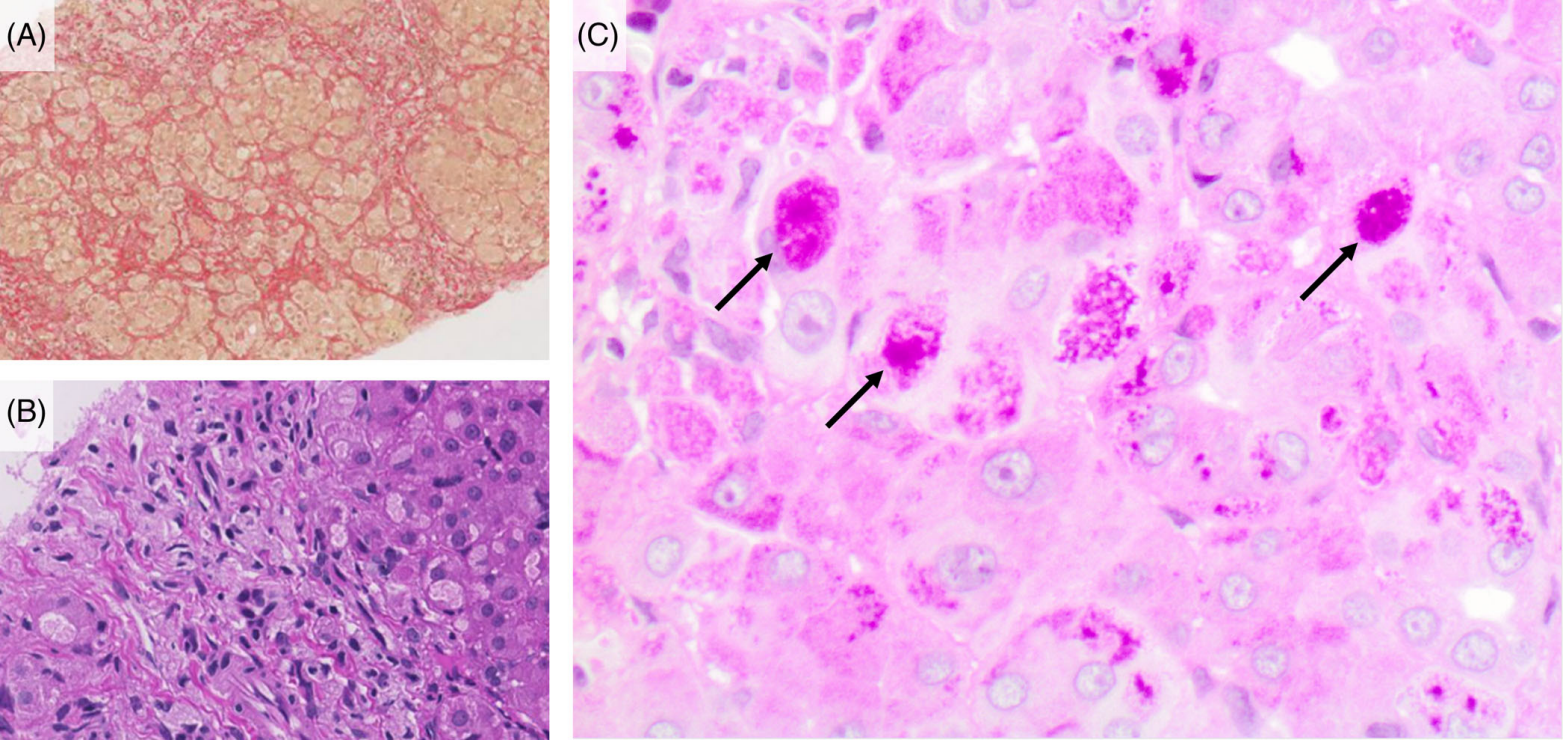
A, Sirius staining (×25): porto‐portal and intralobular fibrosis. B, Haematoxylin eosin staining (×100): pseudolobular liver tissue with broad septae. C, Periodic acid–Schiff (PAS) staining (×400): PAS positive inclusions (arrows), compatible with amylopectin

The GSD IV male patient presented neonatally with failure to thrive, hypotonia, and hepatomegaly. On liver biopsy at 15 months of age glycogen was normal (Figure S1A), but GBE1 enzyme activity was markedly reduced (Figure S1B) and histological studies showed typical amylopectin inclusions (Figure 2). Mutational analysis revealed heterozygous *GBE1* mutations c.1883A > G in exon 14,^12^ resulting in the predicted amino acid substitution p.(His628Arg), and the novel mutation c.2015_2016dup in exon 15, predicted to lead to a truncated protein p.(Ala673ArgfsTer35), lacking parts of the relevant amylase‐like barrel domain.^13^ Multidisciplinary team assessment recommended liver transplantation as the best treatment option, which was performed at the age of 21 months. Subsequently, gross motor skills improved and transaminitis and triglyceride levels (mean of 3.7 mmol/L pre‐ and 0.45 mmol/L post‐transplant) normalized (Figure S1C,D). Urate levels and liver synthesis parameters remained normal throughout (Figure S1E‐G), implying that these are not useful biomarkers in GSD IV disease. BTD enzyme activity in serum was elevated at 15.0 before and normal at 9.0 nmol min/mL after liver transplantation (Figure S1H).

#### 2.2.2 Case 2 GSD Ia

The GSD Ia patient is a female who presented with hypoglycaemic episodes early on in life. The diagnosis was based on significantly diminished G6PC enzyme activity and molecular genetic testing revealing the following compound heterozygous mutations: c.113A > T p.(Asp38Val) and c.562G > C p.(Gly188Arg) in the *G6PC* gene. The clinical course was complicated by the occurrence of an inflammatory bowel disease (IBD)—a rare observation in GSD Ia patients.^14^ The IBD was classified as Crohn's disease and was initially treated with azathioprine and intermittent corticosteroids before infliximab was added to the treatment regime. Throughout her course, metabolic control was suboptimal with continuously elevated lactate (mean of 4.9 mmol/L; reference range of 1.0‐1.8 mmol/L) and triglyceride levels (mean of 8.34 mmol/L; reference range of <2.0 mmol/L) (for both: 9 measurements over a time period of 3 years, 2016‐2019). BTD enzyme activity as assessed over a period of 5 years (2014‐2019) showed an elevated mean of 15.1 nmol/min/mL (SD = 1.5, 13 measurements).

### 2.3 Biotinidase enzyme activity in serum

Enzymatic activity of BTD was assessed in serum of patient and control samples as previously published^11^ by applying a colorimetric assay using biotinyl‐p‐aminobenzoate as substrate. Individual experiments were performed in duplicates and results were expressed in nmol per min and mL serum.

### 2.4 Gene expression studies in liver tissue

For quantitative real time PCR analysis total RNA was extracted from frozen liver tissue sample lysates from the two GSD patients and two controls (QIAmp RNA blood mini kit, Qiagen), amplified to cDNA with the Prime Script II first strand cDNA Synthesis Kit (TaKaRa ClonTech), and analysed for expression using specific probes for *BTD* (Hs00163760_m1), *ACACA* (Hs01046047_m1), *PC* (Hs01085875_g1), *PCK1* (Hs00159918_m1), *PCK2* (Hs01091129_g), *FBP1* (Hs00983323_m1), via the TaqMan Gene Expression Master Mix on a 7900HT Fast Real‐Time PCR System, with normalization to *ACTB* (Hs01060665_g1) (all Thermo Fisher Scientific). Experiments were performed in triplicates.

### 2.5 Statistical analysis

GraphPad Prism 8.0.2 software was used to calculate *t* test and one‐way analysis of variance (ANOVA). Rstudio was used to plot graphs and calculate Pearson's correlation.

## 3 RESULTS

### 3.1 Upregulation of gluconeogenic genes in GSD IV and GSD Ia

**FIGURE 3:**
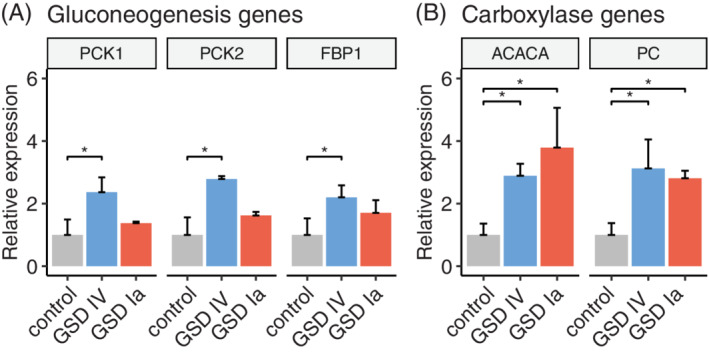
Comparison of expression levels of, A, gluconeogenesis (phosphoenolpyruvate carboxykinase 1 (cytosolic) and 2 (mitochondrial), *PCK1* and *PCK2*; Fructose‐1,6‐bisphosphatase 1, *FBP1*) and, B, carboxylase (acetyl‐CoA carboxylase alpha, *ACACA*; pyruvate carboxylase, *PC*) genes among liver samples from a GSD IV (blue), GSD Ia (red) patient and two control subjects (grey). Bars represent mean relative expression from two independent experiments, each performed in triplicates, normalized to the mean of the control samples. Error bars are SD. Asterisks indicate *P* values <.05 as determined by *t* test for which each gene was analyzed individually

To examine whether gluconeogenesis is affected in GSD IV and GSD Ia, we investigated gene expression levels of key enzymes of gluconeogenesis in liver tissue by RT‐qPCR. The cytosolic phosphoenolpyruvate carboxykinase facilitates the initial step of gluconeogenesis and is encoded by *PCK1*, which was upregulated 2.4 times in GSD IV and 1.4 times in GSD Ia (Figure 3A). The isomer in the mitochondria is expressed from the *PCK2* gene, which showed increased expression in GSD IV (2.8 times) and GSD Ia (1.6 times) (Figure 3A). Upregulation of *FBP1* (GSD IV: 2.2 times; GSD Ia: 1.7 times), encoding fructose‐1, 6‐bisphosphatase 1, which catalyses the hydrolysis of fructose 1,6‐bisphosphate to fructose 6‐phosphate, showed further indication of upregulated gluconeogenesis (Figure 3A). Only the changes observed in GSD IV were significant (*P* values <.05).

### 3.2 Upregulation of carboxylases

To study possible changes of specific biotin‐dependent carboxylases involved in gluconeogenesis and fatty acid synthesis, we examined the expression levels of the *ACACA* and *PC* genes. We hypothesized that the two investigated genes are upregulated as they are required to supply starting substrates for gluconeogenesis (oxaloacetate) and fatty acid synthesis (acetyl‐CoA). Expression of the *ACACA* gene, encoding acetyl‐CoA carboxylase, was upregulated 2.9 times in GSD IV and 3.8 times in GSD Ia (Figure 3B). Additionally, the expression of the pyruvate carboxylase gene was also significantly elevated by 3.1 times in GSD IV and 2.8 times in GSD Ia (Figure 3B).

### 3.3 Biotinidase expression and enzyme activity

To assess the effects of changes in gluconeogenesis and carboxylase genes on BTD, we employed RT‐qPCR in liver and a colorimetric enzyme assay for BTD enzyme activity in serum. We hypothesized that increased levels of *ACACA* and *PC* are accompanied by enhanced BTD enzyme activity to meet increased demands for their biotin cofactor.

**FIGURE 4:**
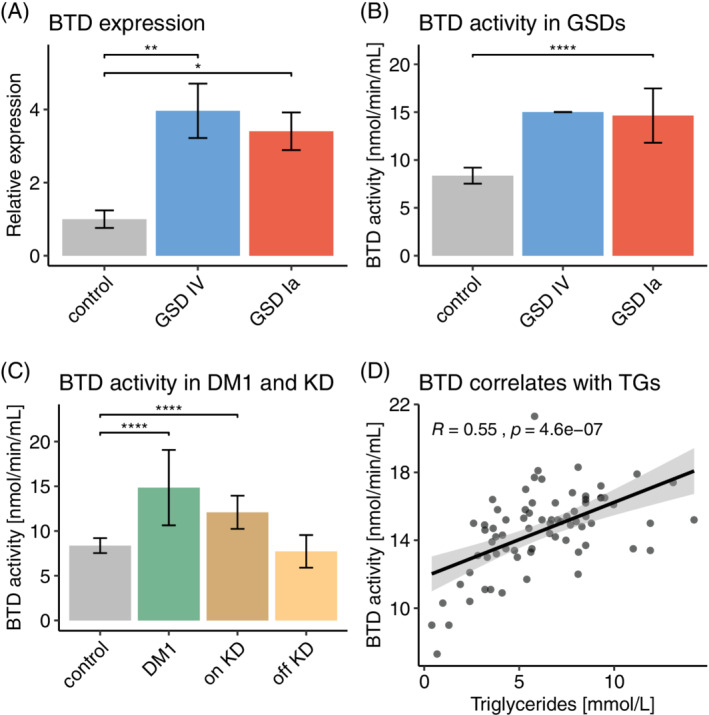
A, Relative expression levels of the biotinidase (*BTD*) gene are higher in liver tissues of a GSD IV (blue) and a GSD Ia (light red) patient compared to two human control liver samples (two independent experiments, each performed in triplicates). B, Biotinidase enzyme activity was assessed in sera of the GSD IV case (blue, assessed in duplicates), GSD Ia patients (red, n = 7 new patients compared with our previous publication^11^), C, diabetes mellitus type 1 patients (DM1, green, n = 5), and individuals on ketogenic diet (on KD, dark brown, n = 4) and the same patients off ketogenic diet (off KD, light brown). The mean biotinidase enzyme activity of control samples (n = 317), assessed from 2011 to 2018, was used to compare with individual patient groups in panels B and C. No comparison was made for GSD IV in panel B, as bar represents only a duplicate measurement. Bars are mean values and error bars indicate SD. Asterisks indicate significant *P* values (* < .05, ** < .01, **** < .0001) as determined by one‐way ANOVA with correction for multiple comparison by Dunnett test

The *BTD* gene was significantly upregulated 4.0 times in GSD IV and 3.4 times in GSD Ia liver (Figure 4A) compared with control. To see if this finding was translated to the biochemical level, we measured BTD enzyme activity in serum and compared it to n = 317 control samples, for which a mean enzyme activity of 8.4 nmol/min/mL with a SD of 0.8 was determined. In GSD IV BTD enzyme activity was significantly increased at 15.0 nmol/min/mL (SD = 0.02, as determined by duplicate measurements) (Figure 4B). Likewise, we confirmed our previous results of significantly increased serum BTD enzyme activity^11^ in a new subset of GSD Ia patients (n = 7; mean enzyme activity 14.8 nmol/min/mL; SD = 2.6) (Figure 4B), including the specific GSD Ia patient whose liver tissue was investigated.

### 3.4 Reversibility of modified biotinidase enzyme activity

To further study whether gluconeogenesis and fatty acid metabolism perturbations play a role in BTD upregulation, we used samples from patients with normal functioning glycogen storage. The first cohort consisted of patients with diabetes mellitus type 1 with reasonable glycemic control (mean HbA1c 9.06%, range 7%‐11.65%), who are known to have increased gluconeogenic flux.^9^ The second group comprised patients on a ketogenic diet, in which elevated levels of acetyl‐CoA activate pyruvate carboxylase.^10^ In DM1 patients (n = 5) the serum BTD enzyme activity was markedly increased to 14.9 nmol/min/mL (SD = 4.7) (Figure 4C), significantly elevated above the normal range. Additionally, a group of epilepsy patients (n = 4), who were on a ketogenic diet as part of the antiepileptic treatment regime, showed an increase in BTD enzyme activity to 12.1 nmol/min/mL with SD of 2.1 (Figure 4C). Interestingly, the raised BTD enzyme activity reversed to normal levels (7.7 nmol/min/mL; SD = 2.1) when the same patients were returning to a standard diet (Figure 4C).

Of note, the BTD enzyme activity level in the presented GSD IV patient dropped back to reference range after liver transplantation (Figure S1H) and his triglyceride levels normalized (Figure S1C). Parallel investigation of triglyceride levels and BTD enzyme activity in a subgroup of GSD Ia patients (n = 5), showed a significant positive correlation of triglycerides and BTD enzyme activity levels, suggesting a direct link between lipogenesis and BTD function (Figure 4D).

## 4 DISCUSSION

Elevated BTD enzyme activity can be used as a biomarker for hepatic GSDs, however, the mechanism of action underlying the increased activity of this enzyme under GSD conditions is unclear so far. In this study, we investigated this phenomenon by analyzing liver and serum samples derived from GSD IV and GSD Ia patients. Together with increased BTD enzyme activity in serum and expression in liver tissue, we detected elevated expression of two biotin‐dependent carboxylases, supporting the hypothesis of increased biotin‐turnover and thus elevated BTD enzyme activity. Similarly, patients with diabetes mellitus type 1 and patients on ketogenic diet, conditions known for increased gluconeogenic flux and activated pyruvate carboxylase, also showed elevated BTD enzyme activity in serum.

### 4.1 Changes in gluconeogenesis and fatty acid synthesis

We have examined four different conditions in this study, namely GSD Ia, GSD IV, diabetes mellitus type 1, and ketosis due to ketogenic diet. Based on published findings and on our results, the four conditions are either linked to increased gluconeogenic flux or increased fatty acid synthesis as follows: (a) In GSD Ia glucose‐6‐phosphate accumulates and is shunted to glycolysis (and the pentosephosphate pathway). This causes increased flux through glycolysis, which leads to raised acetyl‐CoA and consequently malonyl‐CoA.^8^ The latter reaction is facilitated by the ACACA enzyme, in line with our observation of increased *ACACA* transcript levels in the GSD Ia patient liver. (b) In GSD IV the breakdown of pathological glycogen is impaired, in turn requiring an increased baseline gluconeogenic flux to ensure normoglycemia, which is facilitated by increased expression of *FBP1*, *PCK1*, and *PCK2*, as shown in liver tissue of the GSD IV patient. Additionally, *PC* expression is also elevated, enabling the initial reaction of gluconeogenesis. (c) In diabetes mellitus type 1 insufficient endogenous insulin secretion leads to upregulated gluconeogenesis.^9^ (d) In patients on low‐carbohydrate ketogenic diet, oxidation of even‐numbered fatty acids leads to accumulation of acetyl‐CoA, stimulating fatty acid synthesis.^10^

### 4.2 Elevated biotinidase enzyme activity

The above described increase of the *ACACA* gene in the GSD Ia patient liver was also demonstrated in a GSD Ia rat model facilitated by the application of a G6PC inhibitor.^15^, ^16^ While raised *de novo* lipogenesis might not be the only mechanism for hypertriglyceridemia in GSD Ia patients,^17^ the higher abundance of ACACA may lead to an increased demand for biotin and consecutively increased biotinidase levels, which we observed in the GSD Ia liver on transcript level as well as on enzyme activity measurements in GSD Ia patient sera. Similarly, in subjects on ketogenic diet, acetyl‐CoA is increased (Manninen)^18^ and may thereby also lead to an increased need for biotin and subsequently raised biotinidase enzyme activity, as demonstrated by our results.

In the situation of increased gluconeogenesis, as demonstrated in GSD IV by our results of raised key gluconeogenic enzymes on transcript level and as reported in diabetes mellitus type 2 patients,^9^ the primarily increased step is the reaction of pyruvate to oxaloacetate, catalyzed by PC. Together with the increased *PC* transcript level, the *ACACA* gene also shows higher expression in the GSD IV patient liver, possibly due to an effect termed carboxylic acid cycle cataplerosis.^19^

Eventually, our data suggests that as a consequence of the increased levels of the two biotin‐dependent carboxylases ACACA and PC, the demand for their cofactor biotin is raised and *BTD* transcript levels as well as BTD enzyme activity is increased.

Future research should address other factors influencing the pathways discussed in this study, such as effects on intermediary metabolite levels and kinetic activity levels of gluconeogenic as well as fatty acid synthesis enzymes. It will also be of interest to evaluate the correlation between BTD enzyme activity, clinical severity, and episodes of hypoglycaemia in GSD patients. In addition, measurement of biotin levels may be of interest and might provide a rationale to consider supplementation of GSD patients with the biotin vitamin. However, whether a potential normalization of serum biotinidase activity is of any clinical relevance needs to be determined.

Based on the results of our pilot study, we propose that markedly elevated serum BTD enzyme activity in hepatic GSD Ia and GSD IV reflects the imbalance of hepatic glucose metabolism that is caused by (a) accelerated gluconeogenesis and (b) increased fatty acid synthesis, pathways that involve the biotin‐dependent enzymes pyruvate and acetyl‐CoA carboxylase. Future studies aiming at confirming this hypothesis should involve a higher number of samples and could eventually expand the spectrum of BTD enzyme activity as a biomarker beyond GSDs to detect perturbed gluconeogenesis and lipogenesis, such as in diabetes mellitus.

## CONFLICT OF INTEREST

P Forny has no conflicts of interests to declare. P Burda has no conflicts of interests to declare. P Bode has no conflicts of interests to declare. M Rohrbach has no conflicts of interests to declare.

## AUTHOR CONTRIBUTIONS

P. Forny, P. Burda, M. Rohrbach contributed to the concept and design of the study. P. Forny collected patient information, performed RT‐qPCR experiments, interpreted results, and wrote the manuscript. P. Forny serves as the guarantor of the performed study. P. Burda performed biotinidase measurements and revised the manuscript. P. Bode performed histological studies. M. Rohrbach revised the manuscript and provided patient information.

## INFORMED CONSENT

We have obtained written informed consent from the two GSD patients (or their respective caregiver) for their liver samples and blood sera to be analysed and their clinical information to be used. Ethics approval to conduct the study was granted by the Commission for Ethics of the Canton of Zurich (BASEC‐Nr. Req‐2017‐00800). All procedures were in accordance with the Helsinki Declaration of 1975, as revised in 2013. Control liver samples were obtained via the Centralized Tissue‐Biobank of the University Hospital Zurich, Switzerland, for which patients were consented by the biobank team for research usage of their samples. Biotinidase enzyme activity measurements of patients' sera of diabetes mellitus type 1, epilepsy patients on ketogenic diet and GSD Ia patients were performed as part of routine blood testing.

## Supporting information


**Supp. Figure 1** **A.** Glycogen content in liver homogenate assessed as grams per 100 g of liver tissue (ref range: 2.4‐6.4). **B.** GBE1 enzyme activity assessed in liver homogenate (no reference range, compared to intraday control assay). **C.** Triglyceride levels pre and post liver transplantation (2 measurements per mean with a reference range of 0.5‐2.2 mmol/L). **D.** Liver function tests: ALT, alanine transaminase (reference range: <28 U/L); AST, aspartate transaminase (reference range: <35 U/L); GGT, gamma glutamyltransferase (reference range: <23 U/L). **E.** Urate levels in blood pre (*n* = 2) and post (*n* = 4) liver transplantation (reference range: 111‐353 μmol/L). **F.** INR, international normalised ratio (reference range: <1.2). **G.** Albumin in plasma (reference range: 35‐50 g/L). **H.** Biotinidase enzyme activity assessed in serum of case 1 (GSD IV patient) pre and post liver transplantation, ****: *P* < 0.0001 by one‐way ANOVA with Tukey multiple comparisons test. The vertical line in plots D, F and G signifies the time point of liver transplantation at the age of 21 months (Tpx).
