# Supplementary figures and images for: Is serum biotinidase enzyme activity a potential marker of perturbed glucose and lipid metabolism?
Source: JIMD Rep. 2020 Oct 6;57(1):58–66. doi: 10.1002/jmd2.12168 (PMC7802622; doi:10.1002/jmd2.12168)

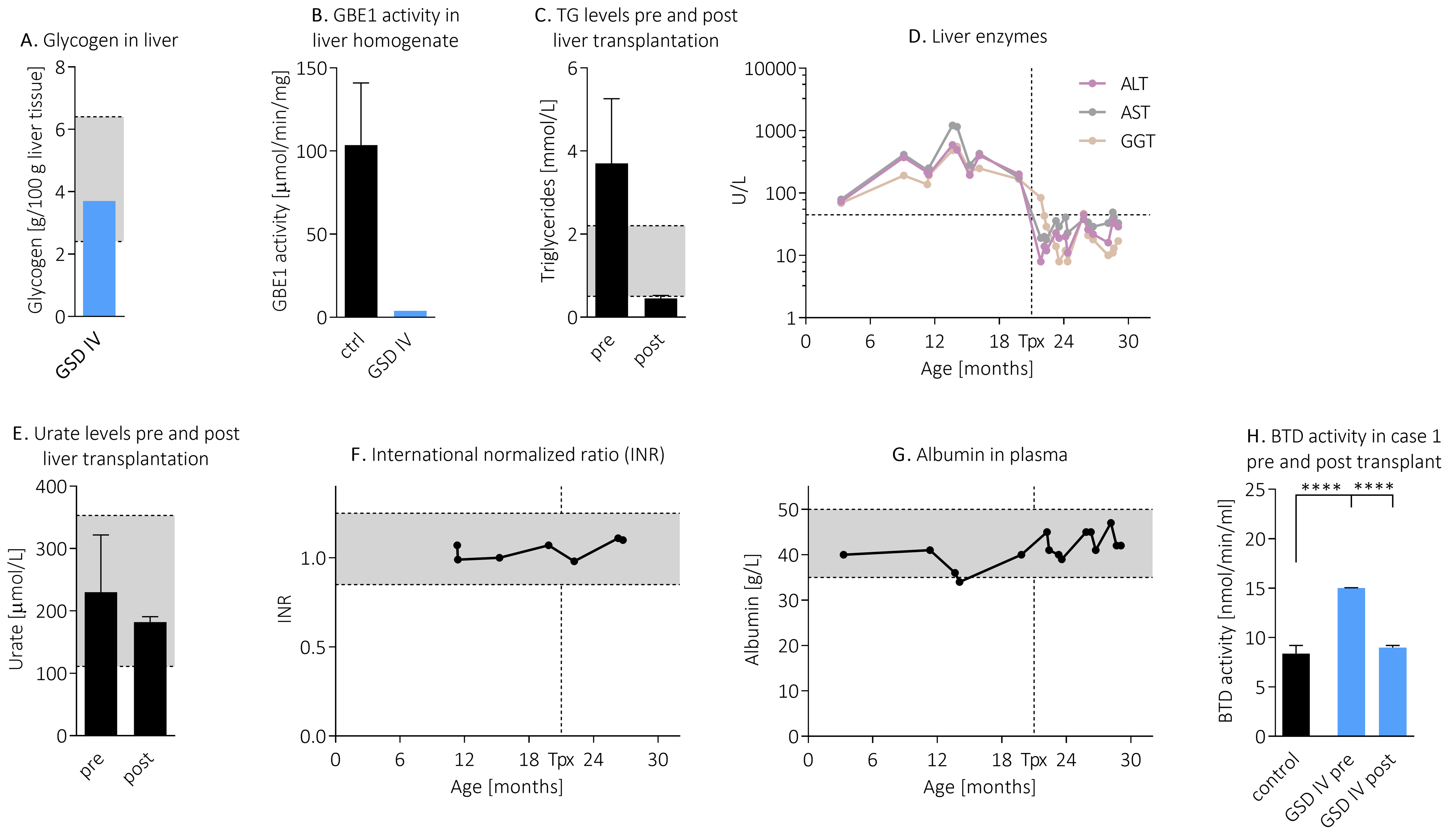

Supplement: Supplementary file 1 — Supp. Figure 1 A. Glycogen content in liver homogenate assessed as grams per 100 g of liver tissue (ref range: 2.4‐6.4). B. GBE1 enzyme activity assessed in liver homogenate (no reference range, compared to intraday control assay). C. Triglyceride levels pre and post liver transplantation (2 measurements per mean with a reference range of 0.5‐2.2 mmol/L). D. Liver function tests: ALT, alanine transaminase (reference range: <28 U/L); AST, aspartate transaminase (reference range: <35 U/L); GGT, gamma glutamyltransferase (reference range: <23 U/L). E. Urate levels in blood pre (n = 2) and post (n = 4) liver transplantation (reference range: 111‐353 μmol/L). F. INR, international normalised ratio (reference range: <1.2). G. Albumin in plasma (reference range: 35‐50 g/L). H. Biotinidase enzyme activity assessed in serum of case 1 (GSD IV patient) pre and post liver transplantation, ****: P < 0.0001 by one‐way ANOVA with Tukey multiple comparisons test. The vertical line in plots D, F and G signifies the time point of liver transplantation at the age of 21 months (Tpx). [file JMD2-57-58-s001.tif]
